# Somatotype, Body Composition and Proportionality in Polish Top Greco-Roman Wrestlers

**DOI:** 10.2478/v10078-011-0031-z

**Published:** 2011-07-04

**Authors:** Katarzyna L. Sterkowicz-Przybycień, Stanisław Sterkowicz, Ryszard T. Żarów

**Affiliations:** 1 Chair of Theory and Methodology of Gymnastics, University School of Physical Education, Krakow, Poland.; 2 Department of Theory and Methodology of Combat Sports, University School of Physical Education, Krakow, Poland.; 3 Chair of Anthropology, University School of Physical Education, Krakow, Poland.

**Keywords:** endomorphy, mesomorphy, ectomorphy, sports level, wrestling

## Abstract

The objective of the paper was to determine body composition and somatotype of male Greco-Roman wrestlers grouped by different weight categories and level of competition. Twenty three contestants (aged 24.9±5.5 years, training experience 13.7±5.8 years) were examined during their competitive period. They were divided into heavier (n=12) and lighter weight categories (n=11).Twelve of them took part in Olympic Qualification Tournaments, whereas six others participated in the Olympic Games in Athens. An experienced evaluator performed 10 measurements necessary to designate Heath-Carter somatotypes and additional skinfolds to estimate the percentage of body fat and body composition. Heavier wrestlers (weight=92.4 kg) exhibited more endomorphy and mesomorphy than lighter wrestlers (weight=70.1 kg). Heavier wrestlers were characterized by higher BMI, fat mass, fat percentage and fat free mass index than wrestlers in lighter weight categories. Sports level was evaluated with discriminant analysis which revealed significant results (p<0.01) with canonical correlation coefficient of 0.754, and Wilks’ λ=0.431. Discriminant function=0.593774*TrainingExperience-0.300177*EN+0.627894*ME-0.242241*EC - 0.636081*Pelvis/Shoulder Ratio. Among the 23 observations used to fit the model, 19 (82.6%) were correctly classified. When compared with untrained subjects, wrestlers exhibited higher body mass (81.8 vs. 72.1 kg, t=3.15, p<0.01) and lower height-weight ratio (40.50 vs. 43.21, t=13.5, p<0.001). Wrestlers’ somatotypes differed from those of untrained subjects (2.0–6.6-1.2 vs. 3.7–4.3-3.1). They were also characterized by lower adiposity (12.1 vs. 15.7%, t=7.84, p<0.001).

In conclusion, body build and composition in wrestlers depend on their weight category. In heavier categories, characteristic type is endomorph-mesomorph, whereas lighter weight categories are dominated by balanced mesomorph. A considerable difference in endomorphy and indices of body composition can also be observed. Higher sport experience with lower endomorphy (tendencies for lower fat content) and Pelvis/Shoulder Ratio are interrelated with higher competition level presented by wrestlers.

## Introduction

Wrestling is numbered among the oldest Olympic sports. Wrestling is characterized as a discipline which makes great demands on athletes in terms of physical preparation. Contemporary Olympic wrestling matches consist of three periods of two minutes each with a 30-second break in between. Athletes must win two of the three periods to capture a match, similarly to tennis and its sets. The total score does not matter. The match format is a change from the 2004 Games in Athens, when wrestlers competed two periods of three minutes each, with a 30-second break in between (Wrestling, 2010). Work time analysis show that mean duration of the matches was 427 s (range 324–535 s), with mean durations of work and rest of 317 and 110 s, respectively. The mean periods of work and rest were 37.2 and 13.8 s, respectively. Mean blood lactate concentration was 14.8 mmol·l^−1^ (range 6.9–20.6). Most of the wrestlers perceived exertion to be highest in the flexors of the forearm, followed by the deltoids and the biceps brachii muscles (Nilsson et al., 2002). The best wrestlers, categorized as elite athletes, are similar in terms of body build and constitute a group which is less differentiated than wrestlers who obtain worse results. They show an exceptionally massive somatic build, characterized by great muscle girths and strongly developed epiphyses adapted to carry higher load (Charzewski et al., 1991). Training experience significantly affected the achievements in strength and strength endurance trials. Body mass affected local endurance of arm and trunk muscles. The sports level clearly differentiated the results of strength endurance of arm and trunk muscles, whose function is extremely important in wrestling (Sterkowicz and Starosta, 2005). In Greco-Roman wrestling, competitors are not allowed to attack their opponent below the waist, nor can they use their own legs to trip, lift or execute other holds (Wrestling, 2010).

Somatotype, which is a synthetic information about body build, is linked to motor abilities. It explains the differences between different disciplines and competitions (Carter and Heath, 1990). There are relationships between somatotype and the level of sports achievement in martial arts (Gualdi-Russo and Graziani, 1993) which was also confirmed in wrestling (Skład et al., 1995). In contemporary competitive sports, research works are gaining in importance, including studies by anthropologists who investigate elite competitors. They allow for obtaining information about somatic determinants for a particular sport. Body mass is largely a function of height. The confirmed dependency of body mass on age, suggesting substantial stability of growth within a development channel, is of essential importance to training practice since it allows for assessment of future target weight categories for adolescent athletes (Tumanian, 1998).

The studies have also found a specific somatotype conducive to being successful in combat sports. Somatotypes typical of elite contestants take a particular surface in a somatogram, determining optimal values in a particular sport. Relationships between body structure and its function are very important and typical of elite level competitors (Claessens et al., 1987). The method of identification of somatotypes (Heath and Carter, 1967) is frequently used for quantitative description of human body build. This method provides information about three components: endomorphy, connected with share of adipose tissue, mesomorphy, relating to muscle mass and ectomorphy, expressed in relationships between body height and weight. Although the above method of description is three-dimensional, individual characteristics of human body build can be also represented in a two-dimensional somatogram (Carter and Heath, 1990).

The literature reports the results of anthropometric studies among athletes, including their somatotype. Carter and Heath (1990) reviewed studies from the sixties, seventies and eighties of the last century and characterized the somatotypes of outstanding senior athletes. The findings concerning body build in elite wrestlers (Carter and Heath, 1990; Igbokwe, 1991; Charzewski et al., 1991; Krawczyk et al., 1997; Skład et al., 1995; Yoon, 2002) are also of much importance. Body build and composition of athletes who took part in competitions of American university leagues have also been investigated (Kanehisa and Fukunaga, 1999; Utter et al., 2001; Utter 2001).

The authors found key factors which determine champion levels, emphasizing the importance of somatic build for specialization in sport (Carter and Heath, 1990 and others). Undoubtedly, updating data which allow for identification and classification of somatic build in top contestants is important for development of a model of contemporary champion in professional sport. Previous studies on athletes in Poland carried out in order to compare somatotypes and body composition have been typically based on a standard of untrained men who study at the Warsaw University of Technology (Piechaczek, 1998). The above literature emphasized differences between wrestlers and untrained subjects in terms of body height. In order to separate the effect of body height in these comparisons, Phantom research tool was developed, with anthropometric dimensions standardized in relation to the model of body height of 170.18 cm (Ross and Wilson, 1974; Ross and Ward, 1982; Ross and Marfell-Jones, 1991). It was repeatedly employed to investigate proportionality of body build in athletes from a number of sports (Collazos et al., 1996; da Silva et al., 2003; Devi, 2006; Keogh et al., 2007; and others).

The aim of this study was to present the body build of Greco-Roman wrestlers in consideration of their weight categories and the level of sports achievement. The following hypotheses were verified:

H1. Somatotype and body composition in wrestlers from heavier weight categories (H) are different than in lighter weight categories (L).
H2: Training experience and body build in competitors are connected with sports level.
H3: Wrestlers are considerably different in body build and body composition than the untrained men.
H4: Morphological profiles reveal characteristic traits of wrestlers, depending on different reference models: (a) untrained students from Warsaw University of Technology (Piechaczek, 1998), (b) Phantom Z-Scores (Ross and Wilson, 1974; Ross and Ward, 1982).

## Material and Methods

### Subjects

During the National Team camp in Polish Olympic Training Centre in Zakopane, in February 2004, a cross-sectional study on 23 Greco-Roman wrestlers in their competitive season was carried out. The data were gathered within a framework of a broader project of PhD thesis (Sterkowicz-Przybycień, 2007) approved by the Council of the Faculty of Physical Education at the University School of Physical Education in Cracow. All interviewed participants were informed about the aim of the study and then agreed to take part in the research.

The subjects were interviewed in order to collect data on age, training experience (in years) weight category and sports level. In the area of the factor of weight category, two levels were distinguished: H – heavier category, i.e. 74, 84, 96, 120 kg (n=12) and L – lighter category, i.e. 55, 60, 66 kg (n=11). The factor of sports level also had two levels (Table 1): I – international (n=12) and N – national (n=11). Wrestlers from group I participated in Olympic Qualification Tournaments, European Championships, World University Championships. Six of them took part in the Olympic Games. Members of group N were not included in the international ranking (Wrestling Data Base, 2004).

### Anthropometric measurements:

Body adiposity was measured by means of a Holtain caliper with a contact surface pressure of 10 g·mm^−2^. In order to determine somatotypes, 10 required measurements were used: body mass (weight scale, model: TBF 300, Tanita Co., Tokyo, Japan) was used for measuring body mass (Wt), body height (measured with anthropometer), four skinfold measurement (triceps, subscapular, supraspinale and medial calf), two girths (arm flexed and tensed, and calf standing), biepicondylar breadths of humerus and femur (Carter and Heath, 1990). Biiliocristal and biacromial breadths have been measured (Sterkowicz-Przybycień, 2007) and proportionality of Pelvis/Shoulder widths ratio was presented. In addition, for the comparison with a group of untrained students (Piechaczek, 1998) the thickness of abdominal skinfold was measured. Data from 165 randomly selected untrained men were used to compare wrestlers’ body build and body composition. Piechaczek also made his results available for skinfold measurements in untrained subjects. A qualified employee of the Department of Anthropology, with a 35-year experience, conducted anthropometric measurements, using the SiberHegner Machines SA (Zurich, Switzerland) instruments. To calculate body density an equation (Piechaczek, 1975):

(1) $$D^{\prime }=1.125180-0.000176\mathrm{LOGtriceps}-0.000185\mathrm{LOGabdominal}$$

was used, with a logarithmic value=100*log10 (compass measurement expressed in tenths of mm minus 18 as the correction for the thickness of the skin). The percentage of fat in body mass was calculated on the basis of the following equation (Keys and Brożek, 1953):

(2) $$\%\mathrm{PF}=100(\frac{4.201}{\text{D}}-3.813)$$

Height-weight ratio HWR (height/mass^−0.33^), body mass index BMI (Wt in kg/height in m^2^), fat mass FM and fat-free mass FFM (Wt-FM) were then calculated. Similarly to BMI, fat-free mass index (FFMI) and fat mass index (FMI) were obtained (Hattori et al., 1997). Those indices were calculated using %PF estimated from Equation 2. In addition, a comparison of %BF assessed with different methods (skinfolds and BIA from Tanita) were presented in the discussion. The evaluation of body composition was performed under standard conditions according to the BIA guidelines(Kyle et al., 2004).

### Statistics

Frequency for weight category (H, L) and competition level (I, N) were compared by means of Chi-square test. Distribution of the number of competitors according to weight category and competition level did not show significant differences (Chi-square with Yates’ correction=0.404, p=0.524). Therefore, competition level demonstrated by the athletes did not depend on weight category (table 1). Mean values (*x̄*) and standard deviation (SD) of age, training experience, height and weight, somatotype (*S̄*) and BMI, FFMI, FMI, and %PF indices were calculated. A computer software ‘Somatotype calculations and analysis’ was used to work out the results pertaining to classification of somatotypes defined by means of the Heath-Carter method (Goulding, 2002). The group average values for both weight category and level of competition were compared by means of the t-test. Somatotype distributions of wrestlers by H and L groups were shown. Individual results in groups of wrestlers were illustrated in a body composition chart (BC), as a single graph which allows for presentation of the BMI, FFMI, FMI, and %PF (Hattori et al., 1997). Discriminant analysis was used to develop a predictive model of group membership with competition level as a grouping factor.

Somatotype of wrestlers, measurements and indices of weight and body composition were compared with a group of untrained subjects (Piechaczek, 1998). Furthermore, profiles (proportionality) of fundamental anthropological measures were mapped for both wrestlers and untrained students by means of Phantom method according to the following formula (Ross and Ward, 1974; Ross and Marfell-Jones, 1991):

(3) Zp-score=1/s*(v*(170.18/h)^d−P)

where: s – is a specified Phantom standard deviation for variable v, v – is the obtained measure of variable v, P=170.18 – is the Phantom stature constant; h – is the obtained stature.

In order to obtain intergroup comparison of wrestlers and untrained men, t-test for independent samples was employed. Data analyses were conducted using the STATGRAPHICS Centurion v. XVI computer software. The level of p<0.05 was considered significant.

## Results

### 

#### a) Comparison by Weight Categories

Table 2 presents characteristics of age, training experience and body build of the wrestlers in consideration of weight category in which they were competing during competitions. Contestants from heavier (H) categories obviously differed from lighter category athletes in body height (t=5.83, p<0.001) and mass (t=5.96, p<0.001). The somatotype of the heavier category showed higher endomorphy than in the lighter category (t=3.72, p<0.01). No significant differences between weight category were found in age, training experience, HWR, mesomorphy and ectomorphy (p>0.05).

**Figure 1:**
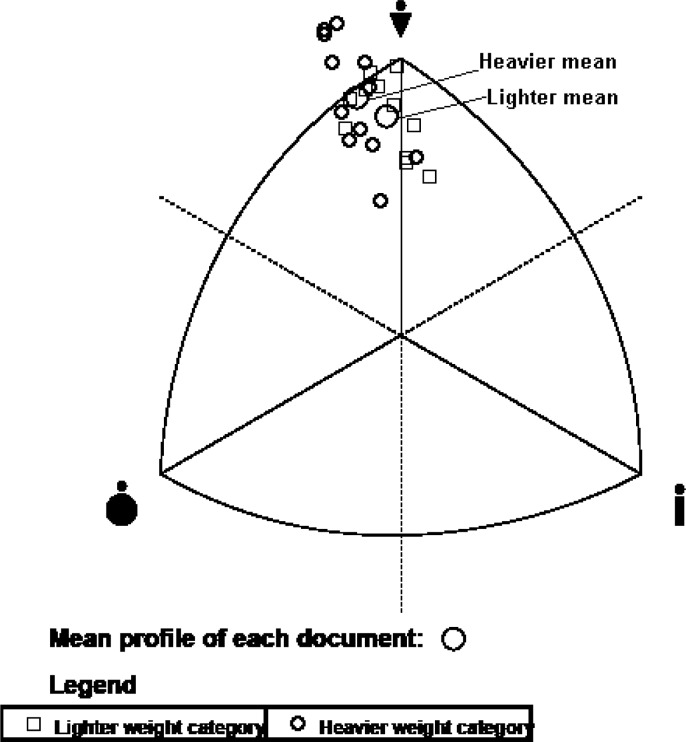
Somatotype distribution of Polish heavier (H) and lighter (L) wrestlers

Figure 1 presents individual somatotypes of wrestlers. Representatives of heavy category was typically (n=10) of endomorphic mesomorph type i.e. mesomorphy is dominant and endomorphy is greater than ectomorphy. Two of them were categorized as balanced mesomorph: mesomorphy is dominant, endomorphy and ectomorphy are lower and do not differ more than by one-half unit. In the group of lighter wrestlers (L), apart from endomorphic mesomorph (n=5) and balanced mesomorph (n=5), ectomorphic mesomorph type was also observed.

**Figure 2:**
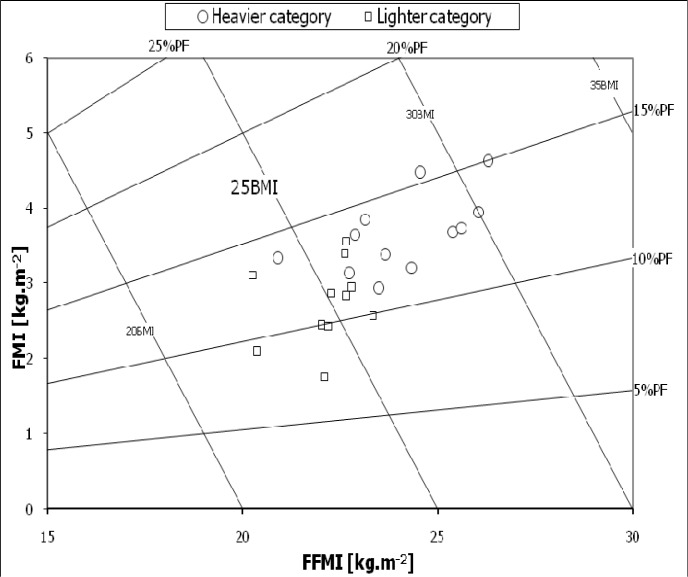
Body composition chart for wrestlers by weight. FFMI – fat-free mass index, FMI - fat mass index. Oblique lines represent BMI – body mass index and %PF – fat percentage in body mass.

Table 3 presents the values of BMI and body composition in the athletes. Significant differences between the representatives of heavier and lighter categories were confirmed for BMI (t=4.22, p<0.001), body composition indices FFM (t= 5.68, p<0.001), FFMI (t=3.53, p<0.001), FM (t=5.52, p<0.001), FMI (t=4.27, p<0.001), D’ (t=3.33, p<0.01) and PF% (t=3.33, p<0.01). Heavier contestants were characterized by the expected higher value of BMI compared to lighter athletes. Body composition of heavier subjects showed not only an advantage of absolute share of FFM and FM but also of FFMI and FMI indices. Their percentage fat (PF%) was significantly higher than in lighter category. Individual characteristics of the studied athletes were presented in body composition chart (Figure 2).

BMI level in category H ranged from 24.2 to 30.9 kg·m^−2^, whereas this value in category L was from 22.5 to 26.2 kg·m^−2^. Most of the wrestlers exceeded a critical value of 24.99 kg·m^−2^, which might point to obesity. Analysis of components of BMI demonstrated that FFMI value ranged from 20.3 to 26.3, whereas FMI amounted to from 1.8 to 4.7 kg·m^−2^. Trained wrestlers showed high FMI indexes in the middle of competitive season, but they were positively correlated with FFMI (r=0.67, p<0.01). Hence, fat percentage in body mass amounted to from 11.1 to 15.4 PF% in the heavier category and from 7.4 to 13.6% in the lighter category.

#### b) Body Build and Composition in Wrestlers in Consideration of Competitive Level

Tables 4 and 5 present body build and body composition in the studied subjects in consideration of their competitive level. Wrestlers of higher level (group I), compared to group N, exhibited higher training experience (t=2.24, p<0.05) and lower endomorphy by 0.4 somatotype units (t=2.15, p<0.05) as well as lower values of Pelvis/Shoulder Ratio (t=3.49, p<0.01).

The discriminant function analysis used the training experience, three somatotype components, endomorphy, mesomorphy and ectomorphy, and Pelvis/Shoulder Ratio by competing level groups. Function 1 is significant (p<0.01) with a canonical correlation coefficient 0.754, and Wilks’ λ=0.431. The coefficient of the function used to discriminate amongst the different wrestling groups is:

$$\begin{array}{l} 0.593774*\mathrm{TrainingExperience}-0.300177*\mathrm{EN}+\\ 0.627894*\mathrm{ME}-0.242241*\mathrm{EC}-0.636081*\mathrm{Pelvis}/\mathrm{Shoulder}\\ \mathrm{Ratio}. \end{array}$$

This function group centroid discriminates between international and national competitors. It separates them by 2.19 units. Two observations in international group were incorrectly classified into national groups. Two observations in national group were incorrectly classified into international group. Amongst the 23 observations used to fit the model, 19 (82.6%) were correctly classified.

#### c) Body Build and Composition in Wrestlers Compared to the Untrained

Tables 2 and 3 also present descriptive statistics for the untrained men. As results from the comparison, wrestlers in total varied in age and were older than the untrained by ca. 4 years (t=9.04, p<0.001). They were characterized by greater body height (t=2.07, p<0.05), greater body mass (t=3.15, p<0.01). Thus, highly significant differences between mean group HWR values (t=13.05, p<0.001) and BMI (t=8.05, p<0.001) were observed, which pointed to more slender body build compared to untrained subjects. Mean BMI in the untrained was within the standard value (≤ 24.99 kg·m^−2^), with higher values in the group of athletes. The value of endomorphy in wrestlers differed from the untrained (t=5.49, p<0.001), similarly to mesomorphy (t=8.83, p<0.001) and ectomorphy (7.47, p<0.001). Mean endomorphy among the wrestlers in relation to untrained subjects was lower by 1.1 somatotype units, ectomorphy: 1.89 units. Advantage of wrestlers in terms of mesomorphy amounted to as much as 2.3 somatotype units. Athletes differed from the untrained in regard to body composition: FFMI (t=9.78, p<0.001), FMI (t=3.00, p<0.01) and PF% (t=7.84, p<0.001), having more FFM and relatively lower fat content (FMI and PF%).

#### d) Profiles of the Trained and Untrained with Regard to Phantom Reference Model

**Figure 3:**
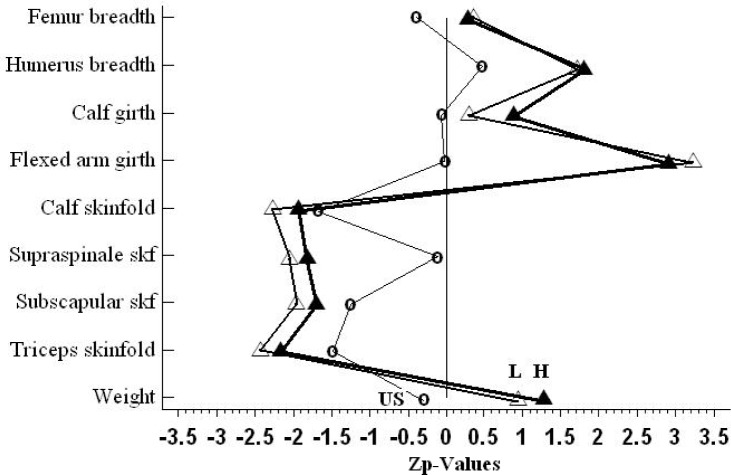
Proportionality profiles for wrestlers (H –heavier weight category, L –lighter weight category) and for untrained subjects (US). Comparison through the Phantom

The abovementioned statistically significant differences in body height in the whole group of wrestlers (W) compared to untrained subjects (US) brought the necessity of profiling their morphological characteristics in the form of Zp-Scores. Data of both heavier and lighter weight categories as proportional scores through the Phantom are presented in Figure 3.

Profiles of wrestlers show considerable similarity of groups H and L. Comparison of anthropological dimensions, necessary for calculation of the somatotype revealed that wrestles in total show high Zp-scores for weight (1.23), humerus breadth (1.76) and especially for flexed arm girth (2.69). At the same time, they are characterized by very high but negative Zp-scores for adiposity (from −1.82 to −2.19). Contrary to the abovementioned anthropologic variables, the Zp-scores of femur breadth (0.34), calf girth (0.60) are close to 0.5. Similarly to the group of wrestlers, US group is characterized by relatively low values of Zp-scores for skinfolds of triceps (−1.47), subscapular (−1.26) and calf (−1.69). In the case of anthropological dimensions: supraspinale skinfold (−0.11), flexed arm girth (−0.03), calf girth (−0.09), humerus breadth (0.45) and femur breadth(−0.38), Zp-scores in US group point to considerable similarity to the Phantom unisex standards.

## Discussion

### Wrestling by weight categories and comparison to untrained subjects

This paper confirms that differences connected with the practiced sport occurred for anthropometric characteristics and indices for competitors of different weight categories. The study also confirmed previous reports (Carter and Heath, 1990) that heavier weight category shows tendencies towards endo-mesomorphy, whereas the lighter weight category, to balanced mesomorphy. Recently, Jagiełło and Kruszewski (2009) found that a characteristic trait of wrestlers in heavier weight categories was also big massiveness of elbow, knee and pelvis width and big diameters of forearm and shin. Moreover, the competitors of heavy weight categories are long-legged. The relatively high values of pelvis width form an average expressed male type of body build.

The characteristics of combat sport competitors compared to untrained subjects available in references are very scarce. A study on the Polish National Team in 1993–1995 (Krawczyk et al., 1997) demonstrated that contestants in Greco-Roman wrestling showed similar body height but greater body mass compared to students, and were characterized by lower value of slenderness index, greater girths in elbow and knee, but similar calf girth. Using a wider comparison (Sterkowicz-Przybycień, 2007) by means of graphical method of profiles standardized for the mean and standard deviation in a group of untrained men (US) (Piechaczek, 1998), a varied level of development of anthropological measures in wrestlers was reported. The wrestlers were shorter compared to the untrained by ca. 0.5 SD and, in consequence, they had lower arm span, length of lower extremities, upper extremities, hands and feet. Shoulder width in the wrestlers was similar to students, whereas a characteristic advantage in the width of the wrist and palm was observed (∼1.5 SD). Considerable differences were also observed for chest, hip, elbow and foot width (>1 SD). Substantial differences between wrestlers and the students were also observed in high, body mass (1.5 SD) at considerably higher arm, forearm, chest and hip girths.

In the present study, differences in measurements of fat, measured either in kilograms or per cents, were lower in relation to the untrained subjects (US). There was a considerable advantage (>1.5 SD) of active tissue (kg, %) in the group of athletes. In consequence, height - weight ratios were higher in untrained subjects. According to Yoon (2002), the range of %PF extends from about 4 to 9% with the exception of super heavy-weights in well-trained wrestlers (Yoon, 2002). More recent studies (Jagiełło and Kruszewski, 2009) showed an increased fat content in heavyweight categories (20%), lower in middle categories (18%) and the lowest in light categories (14.9%). According to these authors (2009), wrestlers of heavy weight categories represent strong type of body build, defined as stout/corpulent and substantially overweight. The notion of overweight might seem disputable. Use of body composition chart (Hattori et al., 1997) by the authors of this study allowed for both individual and group approach to the wrestlers according to weight categories and focusing exclusively on BMI and HWR in elite contestants is insufficient. However, it seems purposeful to concurrently take into account BMI components i.e. FFMI and FMI. Fat percent %PF in the heavier weight category was higher compared to the lighter category. Skład et al. (1995) employed a division into three weight categories, but they did not find similar relationships (Table 6). They emphasized only the low level of PF%. Our study also confirmed significantly lower fat content in the wrestlers in total compared to untrained students (US).

The results of the authors’ investigations of %PF, assessed based on measurements by means of a caliper and BIA method, were similar to previous results of the investigations of Polish National Team (Krawczyk et al.,1997; and Skład et al., 1995 and slightly higher than in Japanese National Team (Kanehisa et al., 1999). Considerably higher fat content was observed in Nigerian National Team (Igbokwe, 1991). Analysis of the results of investigations by other authors reveals that different methods of assessment of %PF were used and only several studies took into account comparison of weight categories (Skład et al., 1995; Carter and Heath, 1990; Sterkowicz-Przybycień, 2007; Jagiełło and Kruszewski, 2009) and comparison to the untrained (Sterkowicz-Przybycień, 2007; Jagiełło and Kruszewski, 2009).

### Sports level

It is also remarkable that, having measurements of biiliocristal breadth and biacromial breadth, the value of proportionality ratio i.e. Pelvis/Shoulder Ratio was additionally calculated. Considerable differences were found between means in groups I and N. Discriminant analysis carried out within this study revealed that higher competition level among wrestlers depended on longer training experience, greater mesomorphy (the musculoskeletal robustness relative to height of physique). At the same time, they exhibited lower endomorphy (the relative fatness of physique), ectomorphy (the relative slenderness of physique) and Pelvis/Shoulder Ratio.

### Somatotype in Time Perspective

For comparisons of the results of the investigations carried out by the authors of this study with available references, a graphical somatogram method (Carter and Heath, 1990) and the analysis of mean (ANOM) were employed. Its advantage lies in determination of the mean for the whole set of data as well as upper and lower decision limits in the chart, which supports interpretation of statistical significance and the direction of mean intergroup differences (Nelson et al., 2005).

**Figure 4:**
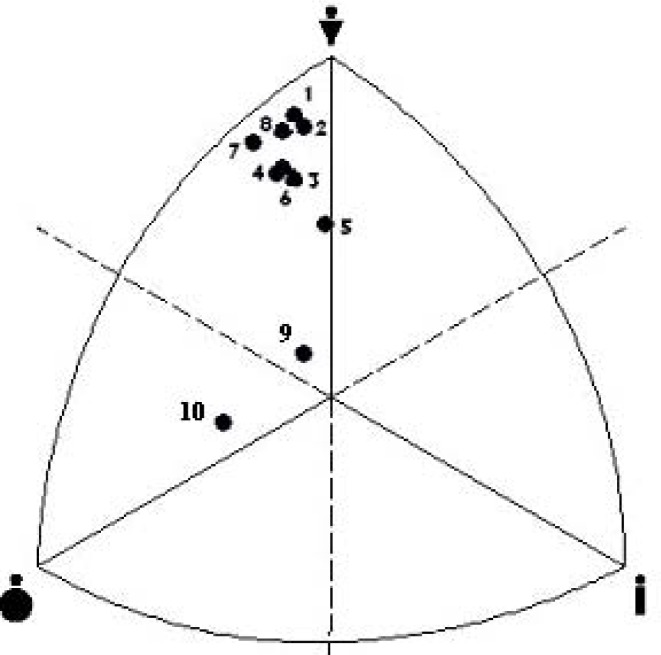
Somatotypes of Greco-Roman wrestlers: 1- POL This study → endomorphic mesomorph; 2- Cuba, Betancourt, 2002 → endomorphic mesomorph; 3-POL 1993-95, Krawczyk et al., 1997 → endomorphic mesomorph; 4-POL seniors 1994, Skład et al., 1995 → endomorphic mesomorph; 5-POL juniors 1994, Skład et al., 1995 → balanced mesomorph; 6-POL 1990, Charzewski et al., 1991 → endomorphic mesomorph; 7- Cuba 1976–80, Rodriguez et al., 1986 → endomorphic mesomorph; 8- Czechoslovakia 1973, Stepnicka et al., 1976. → endomorphic mesomorph; 9-Untrained subjects, Piechaczek, 1998 → endomorphic mesomorph; 10-Phantom, Ross and Ward, 1974 → mesomorphic endomorph.

Figure 4 presents somatotypes of Greco-Roman wrestlers extracted from different studies. The wrestlers typically showed endomorphic mesomorph somatotype. Their characteristics were located in left upper field of the somatogram. Only Polish juniors (#5) were categorized under balanced mesomorph. Furthermore, mean values of components of endo-, meso- and ectomorphy revealed among the contestants in different studies were analyzed by means of ANOM graphical method (Figure 5 A-C).

Although endomorphy in wrestling teams does not statistically differ from grand mean, more recent studies have revealed a tendency towards reduction of share of this component in body build. Mesomorphy in Polish National Team studied in 1993–95 (#3) is the lowest and considerably differs from grand mean. At the level of ectomorphy, none of the studied groups can be distinguished from grand mean. The analysis of the results of the investigations depicts wrestlers as a group where selection of a somatotype is of great importance.

### Which comparison is better?

Use of unisex phantom model (Ross and Ward, 1974; Ross and Wilson 1982) for profiling of body build characteristics in trained Polish wrestlers seems to be disputable. Profiling by means of the abovementioned method revealed, similarly to normalization to the mean=1 and standard deviation=0 in the group of the untrained from Warsaw University of Technology (Sterkowicz-Przybycień, 2007), significant characteristics which result from training and sports selection. It also demonstrated differences (Zp-scores) in comparative group of untrained subjects (Piechaczek, 1998). Zp-scores concerning flexed arm girth and supraspinale skinfold (skinfold patterning) in untrained subjects seem to be striking. We suggest that more updated (in consideration of secular trend) data for the population of the untrained should be adopted for assessment of proportionality in Polish athletes. The question remains ‘whether we should attempt to use them in the equation for Zp-Score suggested by Ross and Wilson (1974)?’

A limitation of this cross-sectional study lies in that BMI cannot provide complex information about the variability of FM and FFM especially in high-level athletes. The methods for assessment of body composition (e.g. regression equation) can be also affected to some degree by the particularity of the observed group. Some solutions are provided by underwater weighing or a newer Bod Pod methodology, but neither of them is portable.

## Conclusions

1. Body build and composition in wrestlers depend on their weight category. In heavier categories, the characteristic type is endomorphmesomorph, whereas lighter weight categories are dominated a by balanced mesomorph. A considerable difference in endomorphy and indices of body composition can also be observed.
2. Higher sports experience with lower endomorphy (tendencies for lower fat content) and Pelvis/Shoulder Ratio are interrelated with higher competition level presented by the wrestlers.
3. Although wrestlers in total, similarly to untrained subjects, were categorized as endomorphic mesomorph, they demonstrate a range of specific characteristics in body build, connected with the demands of training and competition. In somatotype, which is a synthetic approach to body build, a considerable advantage of wrestlers over the untrained occurs in mesomorphy, whereas lower values are observed for endomorphy and ectomorphy. The athletes, who have more FFM, exhibit lower relative indices of fat content compared to the untrained.
4. Selection of a standard comparative group, i.e. untrained Polish students from the Warsaw University of Technology (Piechaczek 1998), or unisex Phantom data (Ross and Wilson 1974, Ross and Ward 1982) might affect the way of drawing conclusions about typical characteristic of body build in Polish athletes.
5. Zp-Scores are suggested to be calculated during assessment of proportionality, using measurements from populations of the athletes and the untrained from Poland.

## Figures and Tables

**Figure 5A:**
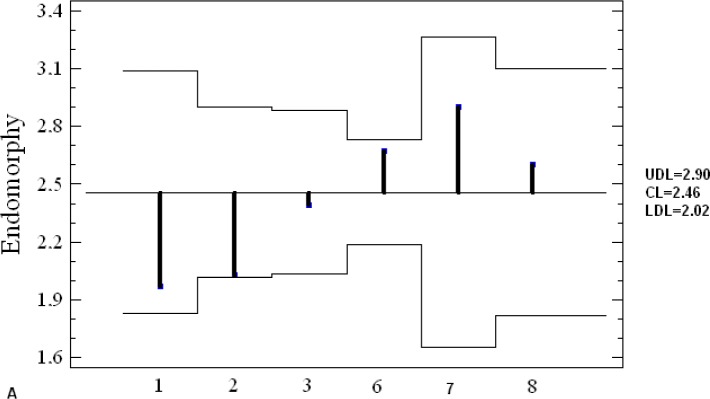
Analysis of means plot of endomorphy with 95% decision limits. UDL - upper decision limit, CL - central line, LDL - lower decision limits

**Figure 5B:**
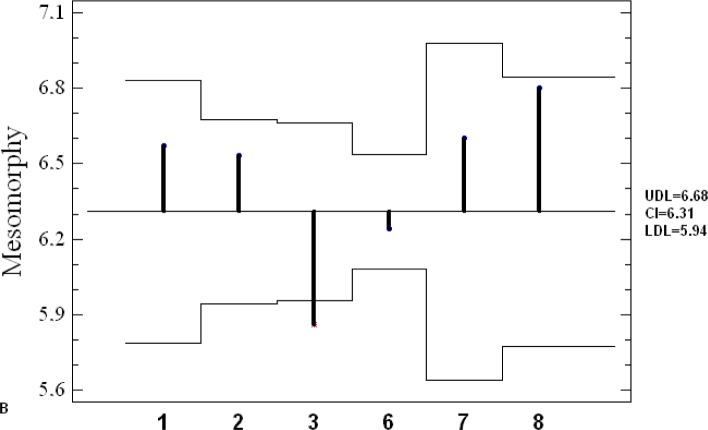
Analysis of means plot of mesomorphy with 95% decision limits. UDL - upper decision limit, CL -central line, LDL - lower decision limits ***5A-C.*** *Mean analysis (ANOM): 1-POL Present study; 2- Cuba, Betancourt, 2002; 3-POL 1993-95, Krawczyket al, 1997; 6-POL 1990, Charzewskiet al., 1991; 7- Cuba 1976-80, Rodriguez et al., 1986; 8-Czechoslovakia1973, Stepnickaet al., 1976*

**Figure 5C:**
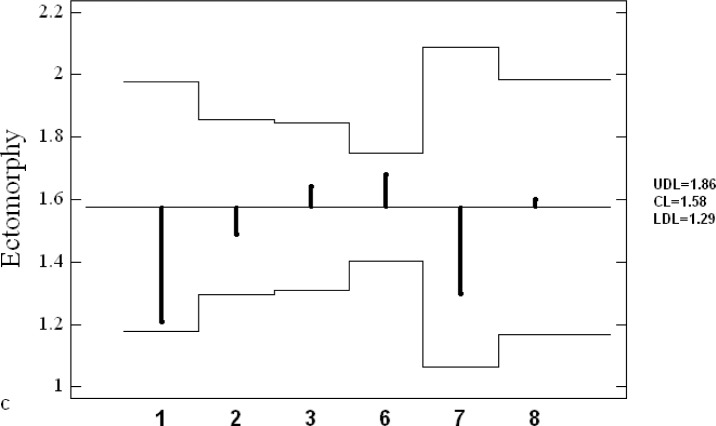
Analysis of means plot of ectomorphy with 95% decision limits. UDL - upper decision limit, CL -central line, LDL - lower decision limits.

**Table 1:** Frequency for competition level and weight categories of 23 top Polish wrestlers

**Groups**	**I**	**N**	**Total**
H	5	7	12
L	7	4	11
Total	12	11	23

*I – International, N – National, H – Heavier weight category, L – Lighter weight category*.

**Table 2:** Age, height, weight and HWR and somatotype of male Polish Team Greco-Roman Wrestlers according to weight category (mean ± SD).

**Weight category**	**Age (years)**	**Training experience (years)**	**Height (m)**	**Weight (kg)**	**HWR**	**Somatotype**		
						**EN**	**ME**	**EC**
H (n = 12)	23.5 ± 4.57	12.0 ± 5.46	1.82±0.06	92.4±10.74	40.36±0.88	2.2 – 6.8 – 1.1±0.3 – 1.0 – 0.5		
L (n = 11)	26.5±6.27	15.5 ± 5.84	1.68^*^±0.05	70.1^*^±6.34	40.74±0.65	1.7^*^ – 6.3 – 1.3±0.4 – 0.7 – 0.4		
Total (n = 23)	24.9±5.53	13.7 ± 5.80	1.75±0.09	81.8±14.29	40.50±0.78	2.0 – 6.6 – 1.2±0.5– 0.9– 0.5		
US (n = 165)	20.6±0.97	.	1.79±0.06	72.1±8.96	43.21±1.66	3.7 – 4.3 – 3.1±1.5– 1.2– 1.2		

US – untrained subjects from Warsaw Technical University (Piechaczek, 1998), HWR = height/weight ratio, or stature/weight^0.3333^, EN – endomorphy, ME – mesomorphy, EC – ectomorphy,

^*^ – indicates statistically significant difference from H, p<0.05.

**Table 3:** BMI and body composition variables for male Polish Greco-Roman Team Wrestlers according to weight category (mean±SD).

**Weight category**	**BMI kg·m^−2^**	**FFM (kg)**	**FFMI kg·m^−2^**	**FM (kg)**	**FMI kg·m^−2^**	**D’ (g ·cm^−3^)**	**PF (%)**	**Pelvis/shoulder ratio**
H (n = 12)	27.8±1.95	80.2±9.11	24.10±1.60	12.2±2.10	3.7±0.51	1.0645±0.004	13.2±1.30	70.0±3.12
L (n = 11)	24.8^*^±1.18	62.4^*^±5.07	22.1^*^±0.97	7.7^*^±1.74	2.7^*^±0.54	1.0711^*^±0.005	10.9^*^±1.89	67.3±3.37
Total (n = 30)	26.3±2.16	71.7±11.63	23.2±1.65	10.1±2.96	3.2±0.70	1.0679±0.005	12.1±1.95	68.7±3.46
US (n = 165)	22.4±2.46	60.6±6.28	19.5±2.02	11.5±3.20	3.7±1.03	1.0580±0.007	15.7±2.74	69.9^a^

US – untrained subjects from Warsaw Technical University (Piechaczek, 1998), BMI – Body mass index, FFMI – Fat-free mass index, FMI – Fat mass index, PF (%) – Percent Fat (%),

^*^ – indicates statistically significant difference from H, p < 0.05,

^a^ calculated from mean biiliocristal and biacromial breadths. Fat percentage in body mass was assessed by means of skinfold method.

**Table 4:** Age, training experience, height, weight, HWR and somatotype of male Polish Greco-Roman Team Wrestlers according to their sports level (mean±SD)

**Sports level**	**Age (years)**	**Training experience (years)**	**Height (m)**	**Weight (kg)**	**HWR**	**Somatotype**		
						**EN**	**ME**	**EC**
International (n = 12)	26.7±5.33	16.1±4.87	1.73±0.10	80.1±16.40	40.42±0.79	1.8 – 6.7 – 1.1±0.5 – 1.0 – 0.4		
National (n = 11)	23.0±5.31	11.1^*^±5.80	1.77±0.08	83.5±12.12	40.68±0.79	2.2^*^ – 6.4 – 1.3±0.3 – 1.0 – 0.5		

HWR = height/weight ratio, or stature/weight^0.3333^, EN – endomorphy, ME – mesomorphy, EC – ectomorphy,

^*^ – indicates statistically significant difference from International, p<0.05.

**Table 5:** BMI, body composition and somatotype variables for male Polish National Greco-Roman Team Wrestlers by their sports level (mean±SD).

**Sports level**	**BMI kg·m^−2^**	**FFM (kg)**	**FFMI kg·m^−2^**	**FM (kg)**	**FMI kg·m^−2^**	**D’ (g·cm^−3^)**	**PF (%)**	**Pelvis/shoulder ratio**
International (n = 12)	26.3±2.55	70.7±13.13	23.3±1.78	9.3±3.39	3.0±0.80	1.0699±0.005	11.4±2.03	66.7±2.18
National (n = 11)	26.4±1.77	72.7±10.28	23.0±1.56	10.8±2.33	3.4±0.55	1.0658±0.004	12.9±1.59	70.8^*^±3.38

BMI – Body mass index, FFMI – Fat free mass index, FMI – Fat mass index, PF (%) – Percent Fat (%),

^*^ – indicates statistically significant difference from International, p<0.05. Body mass was assessed by means of skinfold method.

**Table 6:** Percent fat in body mass for wrestlers

**Autor**	**Country, method**	**n**	**Body mass (kg)**	**% PF**
Present study (Sterkowicz-Przybycień, 2007)	Poland, Greco-Roman wrestlers	23	59.2–109.3	
	Caliper (Slaughter et al., eq. 1)			9.76±2.48
	Caliper (Keys and Brożek)			12.10 ±1.95
	BIA Tanita TBF-300			11.37±2.87
Krawczyk et al., 1997	Poland (National Team) Caliper	51	76.20±10.10	10.3±2.80
Skład et al.,1995	Poland (National Team) Caliper	21		10.37
		.	59.7±3.0	10±1.9
		.	71.0±6.14	8.5±1.4
		.	99.6±17.0	11.9±5.2
Igbokwe, 1991	Nigeria (National Team) Caliper	23	66.5 ± 3.7	18.2 ± 1.8
Kanehisa et al., 1988	Japan Caliper (Keys and Brożek)	33	55–94	10.49±3.11
